# Interrelation of visual and olfactory impairments in schizophrenia

**DOI:** 10.1192/j.eurpsy.2021.434

**Published:** 2021-08-13

**Authors:** M. Tumova, V. Karpinskaya, E. Bezgacheva, L. Muslimova, E. Bigday, M. Ivanov

**Affiliations:** 1 Psychiatry, V.M. Bekhterev National Medical Research Center Psychiatry and Neurology, St. Petersburg, Russian Federation; 2 Neurovisualization, N.P. Bekhtereva Institute of Head RAS, St-Petersburg, Russian Federation; 3 Laboratory Of Olfactory, I.P. Pavlov Institute of Physiology RAS, Saint-Petersburg, Russian Federation

**Keywords:** schizophrénia, visual and olfactory impairments, cognition functions

## Abstract

**Introduction:**

In schizophrenia, there are disorders in all sensory modalities, but the regularities of their occurrence, their pathogenesis and attitude towards cognitive functions are not sufficiently studied.

**Objectives:**

Examine the interrelation between the dysfunctions in different analysers (olfactory and visual) and their dependence on the duration of the disease and the severity of psychotic symptoms and cognitive deficit in schizophrenic patients (F20 according to ICD 10 criteria).

**Methods:**

All subjects were determined the threshold of olfactory sensitivity to n-butanol, the ability to discriminate against odors and the amount of error in comparing the same sections. Cognitive functions were evaluated using the BACS scale.

**Results:**

The inverse correlation between the value of the visual assessment error and the reduction of the threshold of olfactory sensitivity (r=- 0.56; p < 0.05) and the inverse correlation between the value of the visual assessment error and the ability to discriminate smells (0.64; p < 0.05) were revealed. There are no significant correlations between the duration of the disease and sensory disturbances. Olfactory and visual disturbances in schizophrenic patients were connected with cognitive functions ((r=-0,62; p< 0,05 and r=-0,84, p< 0,001 accordingly).

**Conclusions:**

The data confirm that sensory impairments have a common pathogenesis and are closely related to cognitive deficits. Sensory and cognitive deficits in schizophrenia may be the result of top-down regulation failure.

**Disclosure:**

No significant relationships.

